# The role of personalised medicine in treating high-risk neuroblastoma in paediatric patients

**DOI:** 10.3389/or.2026.1850627

**Published:** 2026-10-07

**Authors:** Daniele Ammeti, Debora Curci, Martina Franzin, Marco Rabusin, Melania Eva Zanchetta, Antonella Fabretto

**Affiliations:** 1 Department of Translational and Advanced Diagnostics, Institute for Maternal and Child Health - IRCCS ”Burlo Garofolo” - Trieste, Trieste, Italy; 2 Department of Pediatrics, Institute for Maternal and Child Health - IRCCS “Burlo Garofolo” - Trieste, Trieste, Italy

**Keywords:** neuroblastoma, genomics, metabolomics, proteomics, transcriptomics, personalised medicine, predictive biomarkers, treatment

## Abstract

Neuroblastoma (NB) is one of the most challenging paediatric malignancies, accounting for 15% of childhood cancer-related deaths. It is characterised by a high heterogeneity ranging from spontaneous regression to high frequency of metastatic disease at diagnosis. Although current treatment strategies, including chemotherapy, surgery and radiotherapy, have improved outcomes, recurrence often occurs in high-risk NB patients, thus affecting overall survival. In this context, personalised medicine is emerging as a promising approach taking advantage of predictive biomarkers to refine risk stratification and to predict treatment response, together with the development of innovative treatments for high-risk NB. This review summarises the landscape of biomarkers in NB, focusing on genomic, transcriptomic, proteomic, and metabolomic signatures associated with aggressive disease biology, therapeutic resistance, and clinical outcome. We highlight how improved patient stratification could help to identify actionable targets resulting in innovative therapeutic strategies. These include GD2-directed immunotherapy, CAR-T and CAR-NK cell approaches, and pathway-directed agents targeting mainly ALK, and MYCN-associated dependencies. Ultimately, the transition from standardised protocols to a systematic integration of multidimensional biomarker profiles with next-generation therapies promises to transform the clinical management of high-risk NB. Addressing the biological complexity of NB ensures more effective and less toxic interventions, paving the way for personalised therapeutic approaches aimed at preventing relapse in high-risk cases and significantly improving long-term survival.

## 1 Introduction

Neuroblastoma (NB) represents a major clinical challenge as the most frequent extracranial tumour occurring in childhood. Since it originates from the neural crest progenitor cells, NB is categorised as an embryonal neuroendocrine tumour. It is characterised by the involvement of every site of the sympathetic nervous system, from the adrenal glands, where it occurs in the majority of cases, to the superior cervical, paraspinal, and celiac ganglia (1, 2). Worrisome epidemiologic data report that NB accounts for 15% of cancer-related deaths in children. Beyond its high mortality, it appears as the most common malignancy of the sympathetic nervous system accounting for 97% of cases and represents the most common solid tumour in infancy, with a median age of diagnosis of 17 months (3, 4).

The clinical presentation of NB is extremely heterogeneous and can differ depending on tumour location and extent. Due to the widespread presence of neural crest cells, the sites at which NB manifests can vary. When the adrenal medulla is involved, patients display solid abdominal masses. In contrast, when the superior cervical ganglia are affected, a neck mass, and Horner syndrome (ptosis, miosis, anhidrosis) are observed (5, 6). If NB involves the spinal cord, the common features are cord compression and paralysis (7). In addition to localised clinical features, NB is noteworthy for its enigmatic behaviour, which ranges from spontaneous regression to highly aggressive metastatic dissemination at diagnosis (1, 8). In detail, half of patients present with haematogenous spread at diagnosis, with common sites of metastasis including the bone and bone marrow (56%–71%), lymph nodes (31%), and lungs (3%). Fever, weight loss and fatigue are non-specific clinical symptoms. Furthermore, some patients are asymptomatic and are diagnosed incidentally (9).

To address the clinical complexity, the diagnostic workflow requires a coordinated combination of laboratory testing, metabolic profiling and advanced imaging. In the first instance, complete blood count, prothrombin time and partial thromboplastin time, uric acid, electrolytes, creatinine and liver function tests are required for diagnosis. Furthermore, high levels of ferritin and lactate dehydrogenase may also indicate lower survival rate (10–13). From a metabolic standpoint, it is standard practice to analyse the concentrations of urinary metabolites, such as vanillylmandelic acid (VMA), homovanillic acid (HVA) and dopamine, since these catecholamines present in high concentrations in 90% of paediatric NB (14). Other disease markers consist of plasma-free and total normetadrenaline, metadrenaline and methoxytyramine (15). Once high levels of these biochemical markers have been identified, bilateral bone marrow biopsy is fundamental to confirm the diagnosis and provide important information on tumour stage, in accordance with imaging techniques such as ultrasonography, computed tomography, magnetic resonance imaging, positron emission tomography scans, and metaiodobenzylguanidine scintigraphy (16).

Beyond the initial diagnosis, imaging studies and molecular testing have become necessary tools for patient risk-stratification. To avoid discrepancies in disease staging across the world and to find a standardised classification that is useful for comparison and for making unique treatment decisions, the International Neuroblastoma Risk Group (INRG) Task Force has committed to establish a consensus approach for pretreatment risk stratification, based on clinical criteria and image-defined risk factors (17). Genomic testing has acquired a critically important prognostic value, with oncogene *MYCN* amplification serving as the primary molecular driver of adverse outcomes (18).

The rigorous stratification highlights a marked survival gap between risk groups, underlying the urgent need for personalised therapeutic interventions. General consensus exists regarding the lower survival rate of high-risk NB patients in comparison to low- and intermediate-risk ones. In detail, if the latter have a 5-year survival >90% following chemotherapy and surgical resection; 5-year overall survival for high-risk neuroblastoma is only 40%–50%, even after intensive treatment (19).

The global impact of NB is further compounded by significant epidemiological disparities between different regions. Given the hallmarks of the disease, including its clinical heterogeneity and poor prognosis in high-risk patients, multiple treatment challenges arise (20). NB incidence and mortality have increased from 1990 to 2021, especially in regions with low sociodemographic development index: sub-Saharan Africa and South Asia presented higher burden of disease in comparison to North America and Western Europe, suggesting that levels of medical care and possibility of an early diagnosis could impact on patient’s prognosis (21). Unfortunately, Yue and colleagues predicted global incidence, mortality and Disability-Adjusted Life-Years index will continue to increase until 2035 (21).

To tackle these growing global challenges, modern oncological workflow must shift from empirical protocols towards precision strategies. Current treatments include surgery, chemotherapy, radiotherapy and immune therapy. However, there are no effective curative therapies for patients with recurrence, which highlights the significant clinical challenges that remain. In this context, targeted therapies taking into account risk-groups and interindividual variability, as well as the identification of predictive markers of treatment response, could ameliorate the patient’s outcome leading to a higher survival rate. Great efforts in this scientific field have to be done on high-risk NB, since low- and intermediate NB treatment, consisting in surgical resection and 2–8 cycles of chemotherapy, has proven to be effective in most cases (22). Therefore, the present review will focus on personalised medicine and predictive markers of treatment response in high-risk NB patients.

## 2 Molecular and multi-omics biomarkers in neuroblastoma

The integration of genomic, transcriptomic, proteomic, and metabolomic analyses has considerably broadened our understanding of NB heterogeneity. These approaches have identified both clinically validated biomarkers and emerging molecular signatures that may improve risk stratification, disease monitoring, and the development of personalised therapeutic strategies. Beyond their prognostic value, the ability to predict treatment response represents a major goal of precision oncology. In high-risk NB, reliable predictive biomarkers could improve patient stratification by identifying individuals most likely to benefit from specific therapeutic interventions, thereby supporting more personalised treatment selection and reducing exposure to ineffective therapies. Collectively, multi-omics approaches have revealed molecular features associated with tumour aggressiveness, lineage plasticity, treatment response, and immune evasion, highlighting both established biomarkers and promising candidates for future clinical implementation (Figure 1).

**FIGURE 1:**
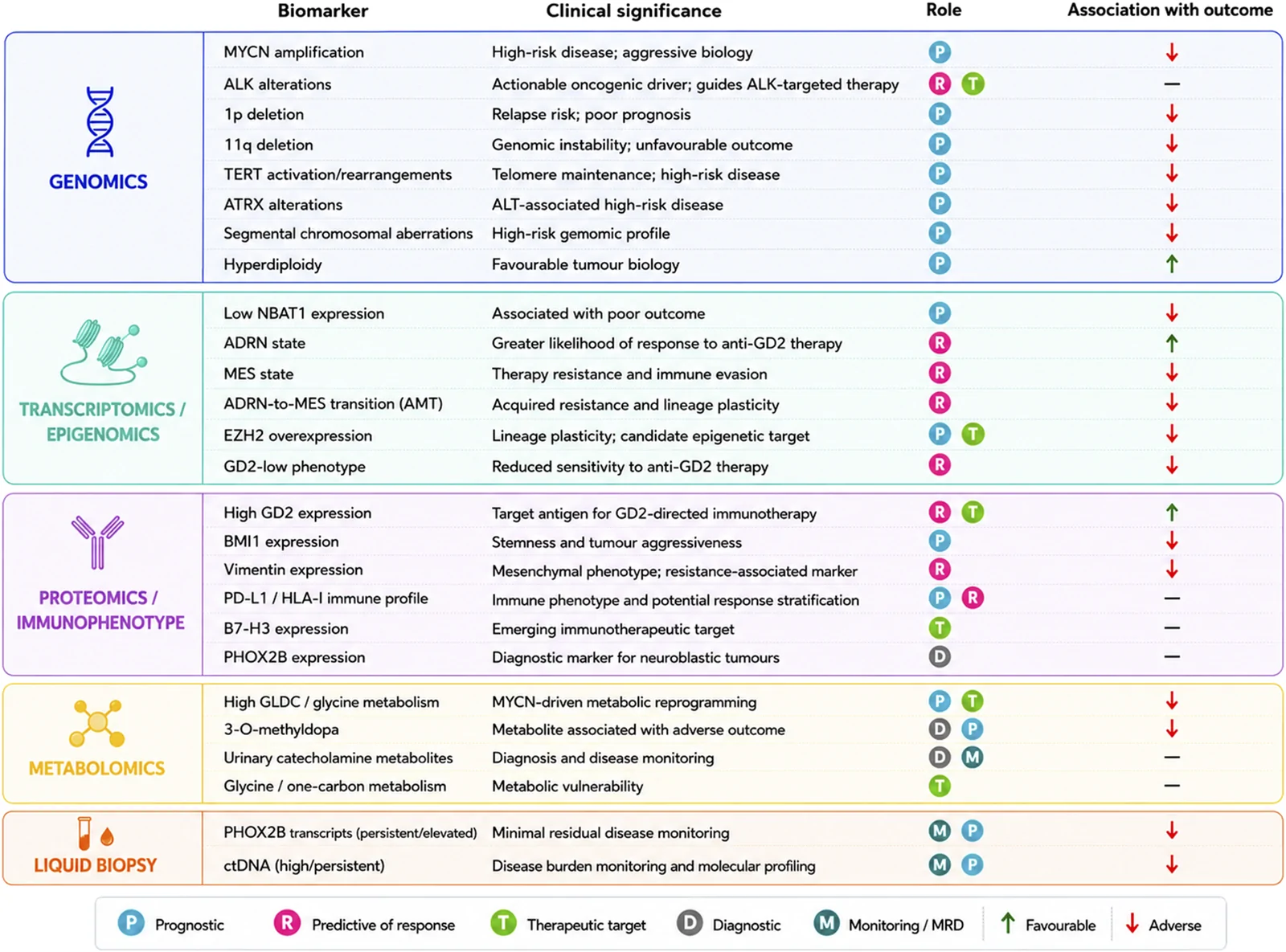
Multi-omics biomarkers and their clinical relevance in neuroblastoma. Biomarkers are grouped into genomics (blue), transcriptomics/epigenomics (turquoise), proteomics/immunophenotype (purple), metabolomics (yellow), and liquid biopsy (orange). P, prognostic; R, predictive of response; T, therapeutic target; D, diagnostic; M, monitoring/minimal residual disease (MRD). Green upward and red downward arrows indicate favourable and adverse outcome associations, respectively; a black dash indicates no established association. ADRN, adrenergic; MES, mesenchymal; AMT, ADRN-to-MES transition; ctDNA, circulating tumour DNA; GD2, disialoganglioside 2.

### 2.1 Genetic alterations and risk factors

Genomic testing in NB operates at different levels of clinical implementation. To date, rapid targeted assays, particularly fluorescence *in situ* hybridisation (FISH), arrays and multiplex ligation-dependent probe amplification (MLPA) for *MYCN* amplification, are incorporated into the diagnostic work-up and contribute directly to current risk stratification (23). In contrast, comprehensive genomic profiling faces severe practical obstacles. Implementing next-generation sequencing (NGS)-based panels, whole-exome sequencing or whole-genome sequencing in routine practice remains challenging. This is primarily due to long turnaround time, high cost, tissue requirements, bioinformatic complexity and the limited immediate actionability of many detected alterations.

However, NGS methods enable large-scale genome studies to identify DNA variations, mutations and expression patterns associated with diseases. This supports precision medicine, allowing the sequencing of individual genomes for personalised therapies. Genomic markers can provide predictive information about how patients with NB will respond to therapies. They can help identify those who may benefit from specific drugs, thereby improving treatment efficacy and reducing side effects. Genomic profiling of NBs revealed that low-stage tumours are characterised by numerical changes in chromosomal copy numbers, whereas high-stage tumours typically exhibit structural chromosomal defects.

Since the 1980s, chromosomal amplification of the *MYCN* proto-oncogene on chromosome 2p24 has been associated with aggressive NB and a poor prognosis (24, 25). It actually occurs in 20%–25% of all NBs so the *MYCN* amplification status remains one of the most critical factors in neuroblastoma risk stratification (26). Mechanistically, MYCN binds directly to the promoter of the Telomerase Reverse Transcriptase (*TERT*) gene, inducing its expression. This explains why NBs with *MYCN* amplification display elevated TERT levels and high telomerase activity. However, some high-risk tumours exhibit high TERT mRNA levels despite the absence of *MYCN* amplification (27).

Genomic rearrangements of the *TERT* locus (TERT-RA) constitute the second most frequent genetic defect in NB (13%–25%). While breakpoints of these rearrangements cluster in a region of approximately 50 kb upstream of the *TERT* transcriptional start site without affecting the gene itself, the chromosomal regions that translocate to the *TERT* locus are highly diverse but uniformly associated with significantly increased TERT expression levels. The presence of TERT-RA is mutually exclusive with *ATRX* and *MYCN* defects, suggesting that these three alterations converge on a common mechanism in high-risk NB. By contrast, *TERT* promoter mutations (TERT-PM), which are frequently detected in other malignancies, are rarely detected in NB but often concurrent with *MYCN* amplification. For these reasons, *TERT* alterations should be considered for the stratification of NB patients: since TERT-RA tumours have elevated TERT expression, the pharmacological inhibition of TERT might improve the outcome for this patient group (26, 28, 29).

Whole-genome paired-end sequencing for 87 untreated primary neuroblastoma tumours of all stages revealed chromothripsis-related structural aberrations and neurogenesis genes alterations that frequently affect genes involved in neuronal growth cones (*PTPRD, ODZ3, ODZ2, CSMD1*) or the regulation of these processes via Rac/Rho signalling (*TIAM1, DLC1, ARHGAP10, ATRX*). All tumours with defects in these genes were aggressive high-stage neuroblastomas (30). Inactivation of the *ATRX* tumour-suppressor gene and *MYCN* amplification are mutually exclusive across all ages and stages in neuroblastoma. Data from mouse models and cell lines support this, showing that the combination of high MYCN expression and loss of ATRX leads to synthetic lethality, likely due to DNA replication stress. An important potential drug target is represented by this, where ATRX function could be reduced in patients with MYCN-amplified tumours, or metabolic (or other) changes caused by MYCN expression in ATRX-mutant tumours could be induced (31).

The list of recurrent somatic genomic alterations identified in NB tumours has grown to include large genomic alterations such as 1p and 11q deletions, unbalanced 17q gain, 19p loss (32).

*ALK* somatic mutations with either point mutations, amplification or fusion events occur in 7%–10% of NB. These mutations result in constitutive phosphorylation and activation of the ALK receptor, thereby leading to aberrant downstream signalling involving pro-proliferative pathways including RAS-MAPK and PI3K-mTOR (33, 34).

Gain of function mutations in the *ALK* gene were also identified as the major cause of highly penetrant, heritable NBs (35). The most common germline *ALK* mutation (R1275Q), which affects the kinase domain and results in constitutive ALK activation, is also the most prevalent somatic *ALK* alteration. Conversely, the most potent somatic *ALK* variants are generally not tolerated in the germline, except for rare cases where NB may co-occur with other neural crest-derived tissue disorders, such as congenital central hypoventilation syndrome (CCHS) and Hirschsprung’s disease (36, 37).

Although most NB cases occur sporadically, approximately 1%–2% are inherited in an autosomal dominant fashion and are considered familial (34). Mutations in the *paired-like homeobox 2B* (*PHOX2B*) gene have been observed in both familial and sporadic cases of neuroblastoma (NB) and represent the first identified genetic predisposition to the disease. Recent functional studies have shown that NB-associated germline *PHOX2B* mutations can block terminal neural crest differentiation in a dominant negative way, thereby facilitating malignant transformation. Furthermore, evaluating PHOX2B expression can serve as a biomarker for distinguishing NBs from other small tumours (38).

Genetic testing for germline mutations in *ALK* and *PHOX2B* is now recommended for children with a family history of NB or those who present with multiple primary tumours. However, the cause of the remaining 10%–20% of familial NBs for which no obvious *ALK* or *PHOX2B* mutations are evident remains to be determined. Also, germline mutations in other cancer-related genes, such as *TP53*, *NF1*, *BRCA1/2*, *NRAS*, *APC*, and *PTPN11*, are considered as predisposing factors (26, 39).

Genome-wide association studies (GWAS) have been used to start defining the genetic landscape of sporadic NB predisposition and have identified common risk alleles in various genes implicated in NB tumorigenesis including *CASC15*, *DUSP12*, *DDX4*, *IL31RA, HSD17B12*, *HACE1*, *LIN28B*, *NEFL*, *CHEK2*, *AXIN2*, *SMARCA4*, *CDKN1B*, *LMO1* and *BARD1.* The most significant and robust association signal identified among them regards *BARD* and *LMO1* in the high-risk subset of NB. These studies suggest that individuals with 'risk' alleles in the *BARD1* and *LMO1* loci are more likely to develop neuroblastoma, and if they do, they are much more likely to experience metastases and worse clinical outcomes (34, 37). *LIN28B* encodes an RNA-binding protein that suppresses the processing of the let-7 family of microRNA (miRNA) precursors and regulates the expression of the oncogenes *RAN* and *AURKA*. This indicates that the LIN28B–RAN–AURKA signalling pathway drives NB oncogenesis, and that this pathway could be targeted for therapy. High LIN28B expression has been shown to disrupt let-7, resulting in elevated MYCN protein expression and promoting NB pathogenesis in mice. Furthermore, LIN28B binds to the promoters of active genes in NB cells through a protein-protein interaction with the zinc-finger transcription factor ZNF143, thereby activating the expression of downstream targets including GSK3B and L1CAM, which are involved in neuronal cell adhesion and migration (1, 40). A genome-scale CRISPR–Cas9 approach was used in a study aimed at identifying clinically relevant molecular markers of cancer dependencies. This study identified *LIN28B* as a candidate gene that is selectively dependent on MYCN-amplified NB tumour cell growth and survival suggesting that therapies designed to inactivate the neoplastic functions of LIN28B may be particularly beneficial for patients with MYCN-amplified neuroblastomas (41).

Recently, gene co-expression network analysis (WGCNA) has revealed the potential role of the *DHFR*, *GMPS* and *E2F3* genes as significant causative drivers of NB. Their high expression has been linked to poor patient prognosis and reduced response to immunotherapy, indicating their potential as prognostic markers (42).

Mutational enrichment analysis revealed a set of active cis-regulatory elements (aCRE), including promoters and enhancers, that had a significantly higher mutation rate and were strongly associated with poorer survival rates in patients with NB. CRE variants were found to modify binding motifs for transcription factors involved in embryonic development and cell differentiation. The expression profiles of many of the IPO7-aCRE, HAND2-aCRE and ARID3A-aCRE target genes, when tested individually and in combination, were associated with markers of poor prognosis and low survival rates. This supports the idea that the non-coding mutational burden has prognostic value, as mutations in aCREs play a biological role in altering the expression of genes involved in neuroblastoma tumorigenesis (43).

Taking everything into account, broad genomic profiling should not currently be considered a routine tool for real-time adaptation of frontline treatment but can contribute to the identification of biologically relevant alterations, candidate therapeutic targets and resistance mechanisms, particularly within clinical trials and in relapsed or refractory disease. In these cases, molecular markers are a valuable tool for patient stratification based on predicted therapeutic outcomes and could potentially accelerate new drug development.

### 2.2 Gene expression-based biomarkers

Transcriptomic profiling has substantially refined biomarker discovery in high-risk NB, in both paediatric and adult populations, by complementing traditional clinico-pathological and genomic risk factors. RNA-seq and high-density expression arrays have enabled the identification of molecular signatures associated with aggressive disease behaviour by capturing genome-wide transcriptional patterns. These signatures include deregulated cell-cycle control, impaired neuronal differentiation and activation of stress-response and immune-evasion pathways. Oberthuer et al. developed and validated a 144-gene prediction analysis of microarrays (PAM) classifier that stratified NB patients into favourable and unfavourable groups with prognostic power independent of conventional risk markers, supporting its use for treatment stratification in high-risk disease (44). Abel et al. further showed that a compact six-gene signature (*ALK*, *BIRC5*, *CCND1*, *MYCN*, *NTRK1*, *PHOX2B*) defines four molecular subgroups with distinct outcomes, underscoring how expression of core lineage and cell-cycle regulators captures aggressive biology (45). More recently, Zhou et al. used Therapeutically Applicable Research to Generate Effective Treatments (TARGET)-NBL RNA-seq data to derive an RNA-Seq Prognostic Index (RPI) and a clinically adjusted RCPI that robustly predicted survival across independent cohorts and outperformed standard risk systems, highlighting the value of genome-wide expression for refining high-risk classification (46). Beyond coding genes, the lncRNA NBAT-1 has emerged as a key transcriptomic marker: Pandey et al. showed that NBAT-1 downregulation promotes proliferation, impairs neuronal differentiation and correlates with poor outcome, and subsequent work and reviews have positioned NBAT-1 and related lncRNAs as central components of NB risk signatures that conceptually extend to rarer adolescent and adult cases (47). In patient samples, longitudinal single-cell and single-nucleus RNA (and ATAC) sequencing before and after induction chemotherapy, as reported by Wienke et al. and Yu et al., revealed therapy-persistent cell states, immune-evasive and mesenchymal-like transcriptional phenotypes and microenvironmental changes that are enriched in patients with a poor response or early relapse. These findings provide dynamic expression markers of minimal residual disease and emerging resistance (48, 49). In parallel, the RNA-seq data from 58 high-risk NB versus normal adrenal tissue samples were reanalysed (50) integrating differential expression with weighted gene co-expression network analysis to define a robust 104-gene core module converging on cell-cycle regulation, DNA-damage repair, neuronal differentiation and oncogenic signalling pathways, and providing a list of biologically annotated genes that can be further investigated as candidate therapeutic targets. Complementary liquid-biopsy RNA-seq studies, including those focusing on disseminated tumour cells and immune-related expression signatures, further support the use of transcriptomic markers not only for baseline risk stratification but also for real-time monitoring of chemosensitivity, guiding early treatment intensification or switch to targeted strategies in high-risk NB (51). These data have uncovered prognostic and response-associated markers beyond classic alterations such as *MYCN* amplification, including specific transcription factors, lineage markers and non-coding RNAs that stratify patients into distinct molecular risk groups; moreover, integrating transcriptomic markers with clinical and genomic data in multi-omic models improves prediction of therapy resistance, metastatic potential and relapse, facilitating more precise risk stratification and the rational selection of intensified or targeted therapies for high-risk disease.

### 2.3 Protein biomarkers

Proteomic profiling offers an advanced functional level of understanding of the biology of NB, going beyond genomic models. A comprehensive analysis of the tumour proteome, aimed at characterising protein expression levels and studying protein degradation and stability, can provide more accurate information about NB response to drugs. However, its role in high-risk NB remains primarily exploratory, although in other tumour types it is increasingly demonstrating potential predictive value (52). Mass spectrometry (MS)-based proteomics offers direct insights into the functional protein landscape, including post-translational modifications, interactions, and localisation. Nevertheless, because of high financial costs and prolonged analytical turnaround times, these analyses are not yet incorporated into routine risk stratification or treatment-selection pathways meaning that MYCN status remains the primary clinical determinant.

When applied in preclinical models, high-throughput proteomics successfully maps the specific molecular networks that drive aggressive behaviour (53). In high-risk NBs, certain proteins, such as those involved in the MYCN signalling pathway or cellular metabolism, can act as biomarkers for predicting drug response. A SILAC approach was used to compare the proteomic signatures of the highly malignant, MYCN-amplified IMR-32 human neuroblastoma cell line and the non-MYCN-amplified SK-N-SH cell line. This revealed the upregulation of proteins involved in cancerous pathways, including leptin signalling, AGE/RAGE signalling, AMPK signalling, the Notch pathway, the SREBP pathway and the Rac1/Pak1/p38/MMP-2 pathway (54). A label-free quantification (LFQ) proteome analysis has also been used to identify 178 differential proteins (107 up- and 71 downregulated) in a SH-SY5Y cell model of neuroblastoma that overexpresses neuroglobin (55). Furthermore, characterisations of aggressive phenotypes have highlighted independent protein drivers of tumour progression; notably, the stem cell renewal factor Bmi-1 has been proven essential for maintaining tumour cell tumorigenicity and stemness, while downstream mesenchymal markers like vimentin closely correlate with cytoskeletal remodelling, increased cell migration, and metastatic potential (56).

The combined density of PD-L1 and HLA class I on tumour cells has been proposed as a prognostic biomarker for overall survival in NB, with high HLA class I and low PD-L1 associated with better outcome (57). More recent data suggest this depends on which cells express PD-L1: on infiltrating NK and CD8^+^ T cells it correlates with favourable outcome, while on tumour cells alone the association is inconsistent across cohorts (58).

The synergistic effect of combinatorial treatment with sirolimus and pyrvinium pamoate on global protein expression was investigated by Yu et al. in a NB cell line that exhibited significant MYCN amplification (SK-N-DZ) using a TMT-assisted LC−MS/MS-based proteomics approach. Functional enrichment analysis revealed that both treatments affected diverse biological processes, with significant differences observed in the proteins that were up- and downregulated in single-drug versus dual-drug treatments. Pyrvinium pamoate influences mitochondrial-related pathways and lipid metabolism, whereas sirolimus affects protein synthesis. Significant downregulation of structural proteins, including ACTB and TUBA1A, was observed. Demonstrating that dual-drug treatment reduces both cell proliferation and the migratory ability of NB cells, as a result of cytoskeletal disorganisation and autophagy-mediated cell death, highlights the potential of combination therapy using the FDA-approved drugs sirolimus and pyrvinium pamoate for treating high-risk NB (59).

Spatial proteomics was employed to validate the intercellular interactions predicted by snRNA-seq in neuroblastoma samples treated with chemotherapy. This was achieved by performing a single-cell multi-omic analysis on patient-matched, longitudinal, high-risk neuroblastoma samples. The results allowed the spatial distribution of neoplastic and immune cells to be determined. After therapy, a decrease in proliferating adrenergic cells, together with an increase in differentiated neuronal-like cells, was observed. Moreover, a higher mesenchymal state at diagnosis was associated with a poorer response to chemotherapy. Conversely, a greater proportion of proliferating and metabolically active neoplastic cells at diagnosis predicted a worse clinical outcome, emphasising the necessity of combinatorial therapies. An increase in the macrophage population after therapy was also observed, probably leading to the activation of the ERK pathway in neuroblasts through HB-EGF upregulation. The ERBB4 receptor, which was expressed at the highest levels on ADRN-like-2 neuroblasts, suggests that ErbB signalling is a pro-tumorigenic mechanism that drives adrenergic neoplastic cells (49).

Telomere maintenance, either by activation of telomerase (as a result of amplified *MYCN* or *TERT* rearrangements) or by alternative lengthening of telomeres (ALT), is a major factor in poor outcomes for NB patients (60). Currently, HR-NB tumours that are ALT-positive are managed clinically with the same treatment protocols as tumours with amplified *MYCN* or *TERT* rearrangements, as they are assumed to have similar biological characteristics. However, large deletions or mutations in the chromatin remodeler *ATRX* gene are also associated with elongated telomeres and ALT in NB (61).

An interesting multi-omics approach aimed at analysing the biology and clinical features of ALT-positive neuroblastoma (NB) highlighted the value of proteomic data in relation to phenotypes, which is closer than transcriptomic data. Many pathways associated with proliferation were significantly downregulated at the protein level in ALT-positive tumours compared to telomerase-activated tumours. Meanwhile, HRAS and NRAS were significantly upregulated together with PAGE5 and MAGEA4, which could be potentially targeted for immunotherapies (62, 63).

Moreover, helicases and chromatin regulators, including ATRX, were less abundant at protein level in the ALT-positive NB, but independently of ATRX mRNA levels and mutation status indicating that reduced ATRX protein abundance is a characteristic proteomic feature of all ALT-positive tumours (either wild-type and mutated *ATRX*) that cannot be observed at the mRNA level which is likely due to the quality control of “orphans” proteins. Consistently, a reduction in DAXX (complex partner of ATRX) protein is also a recurrent event in ALT-positive NB that does not harbour *ATRX* mutations. Therefore, this study provides evidence that patients with ALT-positive NB form a clinically and molecularly distinct subgroup that requires specific treatment, as standard treatment regimens targeting highly proliferating NB are probably unsuitable for treating slowly growing ALT tumours and may induce therapy resistance mechanisms (62).

Proteomic analyses have identified candidate proteins and pathways associated with MYCN status, cellular metabolism and drug exposure, providing a valuable discovery framework for identifying candidate biomarkers, although most available evidence derives from cell-line and preclinical models. In the future, ongoing technological advances and cost reductions may enable proteomics to play a more defined role in NB for biomarker identification and therapeutic stratification.

#### 2.3.1 Exosome proteins as diagnostic and prognostic biomarkers

Extracellular vesicles, including exosomes, represent a valuable source of biomarkers accessible through liquid-biopsy platforms. Their molecular cargo may provide complementary information to tumour tissue analysis since their contents accurately reflect the biological characteristics of the cell of origin.

The characterisation of nanovesicles derived from four human NB cell lines (HTLA-230, IMR-32, SH-SY5Y and GI-LI-N) and the proteomic analysis of HTLA-230-derived exosomes revealed the presence of the pro-inflammatory protein fibronectin, the putative cancer stem cell marker prominin-1 (CD133), the tumour cell-derived collagenase-stimulatory factor basigin (CD147) and the immunosuppressive molecule B7-H3 (CD276) in this cell line, which exhibits high levels of *MYCN* gene amplification. The study also suggests that a signature defined by the presence of the MDK, DBH, CNTN1, NCAM1, CNTFR, PLXNA3 and ADRB2 proteins may characterise cells of neuroblastic origin. Interestingly, strong expression of the GD2 ganglioside, a derivative of cerebroside containing two sialic acid residues that is expressed on most NB tumours and is involved in the inhibition of T, NK and dendritic cell function, was detected in NB-derived exosomes, representing a potential biomarker for NB (64).

Consistently, a recent study characterised the expression profiles of plasma-derived exosomal proteins (exo-proteins) in HR-NB and LR-NB patients, discovering 46 proteins that were upregulated and 46 that were downregulated in HR-NB patients. Upregulation of exosomal NCAM, NCL, LUM and VASP was evident in discriminating NB patients from controls. Furthermore, the upregulation of myosin-9 (MYH9), fibronectin (FN1) and latent transforming growth factor-beta-binding protein 1 (LTBP1), and the downregulation of calreticulin (CALR) and A-kinase anchor protein 12 (AKAP12), were effective in discriminating between HR-NB and LR-NB patients with high accuracy, suggesting their role as diagnostic and prognostic biomarkers (53, 65).

Colletti et al. identified a specific signature that correlates with NB bone marrow dissemination by performing a proteomic analysis of exosomes derived from two primary tumour cell lines and 5 cell lines established from NB bone marrow metastases, as well as 1 cell line derived from a brain metastasis. Exosomes derived from the primary tumour showed upregulation of proteins involved in neuronal processes (development, migration and polarisation), including amyloid precursor-like protein 2 (APLP2), kinesin family member 5C (KIF5C) and tubulins (TUBA1A, TUBB2 and TUBB4B). They also identified proteins involved in ECM organisation and adhesion, such as filamin B (FLNB), laminins (LAMA5, LAMC1 and LAMB1), collagen (COL18A1) and matrix metallopeptidase (MMP15). The expression of proteins involved in mitochondrial activity, such as MRPL4, ECI1, ETFA and ETFB, was increased in NB metastatic cell lines. The proteins that are found only in the exosome derived from the bone-marrow metastatic cell lines are mostly linked to cell survival, growth and cancer development. These proteins include Signal peptidase complex catalytic subunit SEC11 (SEC11A), cell division cycle-associated protein 3 (CDCA3), Nuclear pore complex protein Nup107 (NUP107) and Calcium and integrin-binding protein 1 (CIB1). This comparative proteomic characterisation provides a preliminary basis for identifying predictive biomarkers that could be used to detect and monitor metastatic bone marrow spread in NB (66).

### 2.4 Metabolites as prognostic biomarkers

A critical unmet field in personalised medicine is represented by the prediction of response to therapy in high-risk NB to tailor therapeutic regimens to patients and guide clinical decisions (26). Recent advances in metabolomics, using high-resolution mass spectrometry-based approaches, are increasingly enabling the assessment of metabolic profiles of individuals affected by a disease discriminating at the baseline among responders and non-responders to treatment: this could unveil promising new biomarkers that could stratify patients by their likelihood of responding to first-line therapies or requiring alternative regimens. Combining metabolomics with genomics, transcriptomics and proteomics studies will result in a significantly improved understanding of the interindividual variability in response to therapy (67).

In the context of high-risk NB, the first study investigating the prognostic power of plasma metabolomics (110 patients) conducted by Quintás and colleagues, differences in plasma metabolic profiles between high-risk and low-risk NB patients at diagnosis have been highlighted, enabling to discriminate against high-risk patients with active disease from those in remission. Notably, high-risk NB patients who progressed during treatment exhibited a specific plasma metabolomic signature at diagnosis (68).

Interestingly, Barco et al. conducted the largest work investigating potential new biomarkers of poor prognosis in high-risk NB (172 patients) (69). In detail, they performed untargeted metabolomics analysis based on high-resolution mass spectrometry on plasma samples derived from a first cohort of NB patients and the subsequent differential expression analysis highlighting several metabolites annotated with a high degree of identification. Among these, 3-O-methyldopa, a metabolite related to L-DOPA degradation, was validated in a second cohort of NB patients in a targeted approach: the elevated expression of 3-O-methyldopa correlated significantly with worse survival in patients older than 18 months with metastatic disease (69). Even if further studies on other cohorts of NB patients are needed to confirm the potentiality of 3-O-methyldopa as biomarker of response, in the future this metabolite could be incorporated in clinical panels at diagnosis to better identify high-risk non-responders earlier in the treatment course.

A recent work by Matser and colleagues underlined the clinical significance of urinary catecholamine metabolites, markers widely used for NB diagnosis, in assessing response and complete remission. These results were obtained on a large cohort of NB patients from 2005 to 2021 (70).

In order to understand the molecular mechanism underlying *MYCN*-amplified NB, a comprehensive analysis integrating metabolomics and transcriptomics was conducted by Du et al. (71). In detail, results from the metabolomics analysis of 28 *MYCN*-amplified and 68 non- *MYCN*-amplified NB patients exhibited alterations of 46 metabolites, with 21 displaying elevated levels and 25 demonstrating reduced levels in MYCN-amplified NB. Based on these results, the authors identify a metabolic pattern of high-risk NB with significant alterations in glycerolipid metabolism, purine metabolism, amino acid metabolism (71). In addition to plasma and urinary markers, local tumour metabolic reprogramming provides targetable vulnerabilities; for instance, metabolism-related markers such as glycine decarboxylase (GLDC) have been identified as crucial metabolic drivers in high-risk NB and proposed as novel biomarkers for targeted therapeutic protocols (72).

Despite proven evidence in identification of new metabolites as markers of treatment response, there is the need for large-scale validation of the acquired results, standardisation of metabolomic workflows, and integration with multi-omics and clinical data. However, the current studies support the fundamental role of metabolomics as a promising approach to identify novel predictive biomarkers for high-risk NB treatment response. Furthermore, the main limitation of the presented studies consists in not defining which treatment (chemotherapy, radiotherapy, etc.) patients respond or do not respond to, probably due to the rarity of the disease and therefore to the low sample size.

### 2.5 Liquid biopsy and minimal residual disease monitoring

Longitudinal monitoring of minimal residual disease (MRD) represents a key relevant application of personalised medicine in high-risk NB. MRD refers to subclinical tumour cells that persist below the detection threshold of conventional imaging and standard bone marrow assessment. Because traditional immunocytological examination often lacks the necessary sensitivity, molecular screening has emerged as a far more sensitive tool to quantify residual tumour burdens and predict treatment response (73).

Currently, molecular MRD detection in neuroblastoma mostly relies on quantitative RT-PCR testing bone marrow aspirates. By using multi-marker panels that target neuroblastoma-specific transcripts, including *PHOX2B*, *TH*, *DDC*, *CHRNA3* and *GAP43*, it is possible to detect disseminated tumour cells at levels undetectable by standard techniques (74). Among these, *PHOX2B* stands out for its exceptional specificity.

Clinical data confirm that rapid clearance of bone marrow disease during induction therapy strongly correlates with favourable outcomes, whereas persistent transcript positivity serves as a powerful prognostic marker for high relapse risks and inferior survival (75, 76).

Blood-based liquid-biopsy approaches may further reduce the burden associated with repeated bone marrow sampling and provide information on tumour heterogeneity and clonal evolution. It is particularly useful to assess the presence of circulating tumour DNA (ctDNA), which can be used to detect *MYCN* amplification, *ALK* alterations, chromosomal copy-number changes, methylation markers and patient-specific genomic variants. Serial ctDNA profiling has demonstrated the ability to identify dynamically evolving and potentially actionable genomic alterations during therapy and at relapse, together with its early detection (77, 78). However, low ctDNA abundance during minimal disease, inter-patient variation in tumour DNA shedding and the absence of a universal neuroblastoma-specific genomic marker remain important limitations.

Future studies must prospectively integrate molecular monitoring at key clinical milestones. It is essential to track these markers from diagnosis through induction, consolidation, maintenance, and long-term surveillance. To validate this approach, clinical trials should directly compare molecular data with traditional imaging and bone marrow response criteria.

Before MRD can drive treatment escalation, de-escalation or therapeutic switching, prospective interventional trials must demonstrate that acting on molecular persistence directly improves clinical outcomes. At the bone marrow level, combining sensitive molecular assays with immunotherapeutic targets screening will provide a more precise map of residual disease (79). Ultimately, since liquid biopsies in paediatric solid tumours are still in an early translational phase, they should complement rather than replace established diagnostic standard methods (80).

Therefore, molecular MRD stands out as a powerful, dynamic biomarker that effectively integrates with baseline risk factors, including *MYCN* amplification, by capturing the precise depth and kinetics of treatment response.

## 3 Conventional treatments for patients with high-risk NB

According to the Society of Paediatric Oncology European Neuroblastoma, conventional treatment of high-risk NB consists of a multimodal approach involving induction, consolidation with high-dose chemotherapy (HDC), autologous stem cell rescue (ASCR), local treatment (surgery and/or radiotherapy) and maintenance therapy (81). This therapeutic regimen aims to eradicate cancer as much as possible, limiting the onset of severe toxicity thus leading to long-term survival for this cluster of patients (Table 1).

**TABLE 1:** Conventional multimodal treatment approach for high-risk neuroblastoma.

Step	Treatment	What it involves	When/why it is used	Refs
**1. Induction**	Rapid COJEC	3 cycles of cisplatin, vincristine, carboplatin, etoposide and cyclophosphamide. Given with G-CSF at 10-day intervals	Initial treatment to achieve the greatest possible tumour reduction	(82)
**2. If response is inadequate**	TVD	Topotecan, vincristine and doxorubicin	Used to improve eligibility for the following treatment phases	(83)
**3. Consolidation**	High-dose chemotherapy	CEM (carboplatin-etoposide-melphalan) or Bu-Mel (busulfan-melphalan). Bu-Mel was superior to CEM in HR-NBL1/SIOPEN in children with an adequate induction response and acceptable toxicity	Given after induction to consolidate disease control	(84)
**4. Marrow recovery**	Autologous stem cell rescue (ASCR)	The patient’s previously collected stem cells are reinfused after high-dose chemotherapy	Restores bone marrow function after myeloablative treatment	(85)
**5. Local treatment**	Surgery	Complete excision of the primary tumour, aiming to minimise morbidity	Preferably after induction and before consolidation; improves local control and survival	(86)
**6. Local treatment**	Radiotherapy	Hyper-fractionated radiotherapy (21 Gy). IGRT may use reduced margins and a boost for residual lesions ≥1 cm^3^	After intensive chemotherapy and maximal surgical debulking, to improve locoregional control	(87,88)
**7. Maintenance**	Immunotherapy + differentiation therapy	Dinutuximab beta combined with 13-cis-retinoic acid	Aims to eliminate residual disease and prolong remission; generally well tolerated in the reported study	(89)

Abbreviations: ASCR, autologous stem cell rescue; Bu-Mel, busulfan-melphalan; CEM, carboplatin-etoposide-melphalan; G-CSF, granulocyte colony stimulating factor; IGRT, image-guided radiotherapy; TVD, topotecan-vincristine-doxorubicin.

Achieving maximum tumour reduction is the main purpose of induction chemotherapy. An intensive chemotherapy protocol, consisting of three cycles of cisplatin [C], vincristine [O], carboplatin [J], etoposide [E], and cyclophosphamide [C] (the so-called rapid COJEC regimen), coordinated with granulocyte colony stimulating factor (G-CSF) to reduce toxicity, with a 10-day interval is commonly used (82). Additional cycles of combination of topotecan-vincristine-doxorubicin (TVD) are considered useful for those patients who do not respond adequately for improving subsequent phases eligibility (83).

As previously mentioned, consolidation involves high doses of chemotherapeutic drugs in combination, such as carboplatin-etoposide-melphalan (CEM) or busulfan-melphalan (Bu-Mel). The result from the HR-NBL1/SIOPEN trial showed the superiority of Bu-Mel versus CEM as high dose chemotherapy. Accordingly, an improved event-free survival has been reported in children with high-risk NB having an adequate response to induction treatment and acceptable toxicity profile. In the ongoing trial HR-NBL2/SIOPEN a tandem autologous transplant with HD Thiotepa and BU-Mel is compared with one single transplant with Bu-Mel (84).

ASCR, which involves the reinfusion of a patient’s own collected and stored stem cells back into their body to restore bone marrow function suppressed by high-dose chemotherapy, follows the consolidation phase (85).

Moreover, complete excision of the primary tumour with minimal morbidity through surgery is performed preferably after induction chemotherapy and before consolidation phase. Holmes et al. described that surgical intervention causes improvement in survival and local control (86).

Following intensive chemotherapy and maximal surgical debulking, hyper-fractionated radiotherapy, delivering 21 Gy results in optimal local control (87). Recent studies evidenced that highly-conformal image-guided radiotherapy (IGRT) with reduced margins and a boost dose for residual lesions ≥1 cm^3^ demonstrated excellent locoregional control, comparable to modern literature (88).

Lastly, maintenance therapy, consisting of immunotherapy and differentiation agents, enables the achievement of minimal residual disease and prolongs remission. There is clinical consensus on the administration of the anti-GD2 monoclonal antibody dinutuximab beta in combination with 13-cis-retinoic acid. A recent study by Wieczorek et al. showed that this treatment is generally well tolerated, including in patients with co-morbidities and those who had experienced toxicities with previous therapies (89).

Despite the intensive and multimodal treatment, the prognosis for patients with high-risk NB remains challenging, demanding the need for innovative and potentially more effective therapies. Personalisation of therapy for high-risk NB patients allows for addressing inter-individual differences in therapeutic response, thereby improving the odds of success compared to standard non-adapted conventional treatment.

## 4 Innovative treatments for high-risk NB

The constraints imposed by conventional therapeutic modalities, including surgery, chemotherapy and radiotherapy, in the management of advanced high-risk NB, have made it imperative to develop targeted therapies informed by tumour molecular and genetic characteristics. Understanding tumour biology enables a personalised approach to be developed to counteract the aggressive nature of neuroblastoma. Selective disruption of oncogenic signalling pathways or enhancement of immune-mediated tumour destruction is the primary aim of targeted therapies, including cell-based and immunotherapies, monoclonal antibodies and kinase inhibitors or degraders. The clinical maturity of innovative therapies for high-risk neuroblastoma varies considerably. GD2-directed strategies are already well established in clinical practice, whereas approaches targeting major oncogenic drivers, including ALK and MYCN-associated pathways, and cellular therapies remain less widely available or are still under early clinical or preclinical investigation.

### 4.1 Targeted molecular therapies

Developing targeted molecular therapies that precisely address altered oncogenic pathways is crucial to increase survival rates in high-risk NB, as conventional small molecules approaches often remain ineffective. The molecular therapies under investigation should be considered according to the biological target, the molecular or phenotypic feature used for patient selection, the available therapeutic strategy, and its level of clinical development and span all stages of translational research, ranging from phase III clinical trials to preclinical development (Figure 2) (90).

**FIGURE 2:**
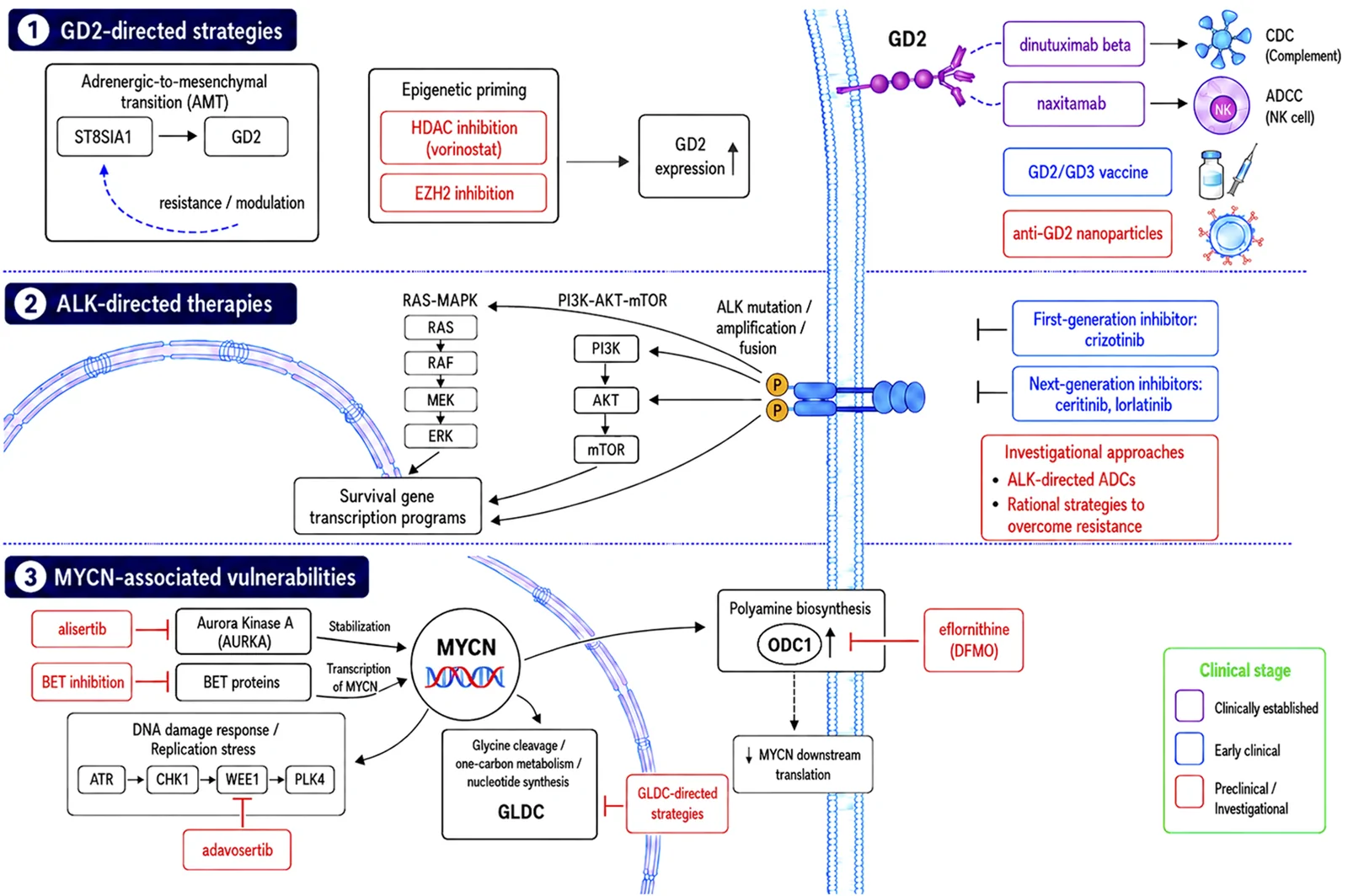
Targeted therapeutic strategies in high-risk neuroblastoma. 1) GD2-directed approaches include modulation of GD2 expression, epigenetic priming, anti-GD2 antibodies, vaccination, and nanoparticles. 2). ALK alterations activate RAS-MAPK and PI3K-AKT-mTOR signalling and can be targeted using inhibitors, antibody - drug conjugates, and combination strategies. 3) MYCN-associated vulnerabilities include MYCN stabilisation and transcription, replication-stress pathways, and GLDC- and ODC1-dependent metabolism. Purple, blue, and red borders around interventions indicate clinically established, early-clinical, and preclinical/investigational strategies, respectively; black boxes represent biological processes. Solid arrows indicate activation or pathway flow; blunt-ended lines indicate inhibition; dashed arrows indicate indirect or feedback effects, whereas dashed connectors provide visual linkage. ↑/↓, increase/decrease; P, phosphorylation. Dotted blue lines separate the panels. ADCC, antibody-dependent cellular cytotoxicity; CDC, complement-dependent cytotoxicity; DFMO, difluoromethylornithine; NK, natural killer cell.

#### 4.1.1 GD2-directed strategies

To date, the most successful approach relies on direct targeting of GD2 - a disialoganglioside highly expressed on most NB cells, although with relevant inter- and intratumoural heterogeneity. Monoclonal antibodies such as dinutuximab and naxitamab (91, 92), combined with standard chemotherapy, have significantly increased event-free and overall survival, becoming the off-label standard of care for high-risk NB in several countries (93–97). Reinforcing this therapeutic importance, the recent Paediatric Strategy Forum on GD2-targeting agents emphasised the need to standardise GD2 expression assessments, compare available anti-GD2 platforms and prioritise rational combination strategies that improve efficacy while limiting on-target, off-tumour toxicities, particularly neuropathic pain (98).

Another promising strategy involves administering the bivalent GD2/GD3 vaccine, which is currently being tested in Phase II trials to stimulate a robust immune response against the tumour and potentially increase patients’ 5-year survival rates (99, 100).

Despite the clinical benefit of anti-GD2 monoclonal antibodies, tumour-cell plasticity is a relevant mechanism of resistance that may constrain durable responses. Specifically, their transition from an adrenergic to a mesenchymal-like state (AMT), associated with increased expression of mesenchymal markers such as BMI1 and vimentin, can reduce surface GD2 expression through downregulation of ST8SIA1 (GD3 synthase), resulting in resistance to anti-GD2 antibody-mediated cytotoxicity. This resistance mechanism has been addressed in preclinical models through pharmacological inhibition of EZH2, a histone-lysine N-methyltransferase, with tazemetostat, promoting epigenetic reprogramming towards an adrenergic-like state, thus restoring ST8SIA1 and GD2 expression, and enhancing sensitivity to dinutuximab (101). However, no registered clinical trial has yet selected patients on the basis of a GD2-low/mesenchymal phenotype or tested an anti-GD2 antibody in combination with tazemetostat. This is largely due to critical safety warnings regarding EZH2 inhibitors, therefore clinical translation will require EZH2-targeting agents with an acceptable long-term safety profile.

Histone deacetylase (HDAC) inhibition may provide a complementary epigenetic strategy to enhance GD2-directed immunotherapy. In an orthotopic neuroblastoma model, the HDAC inhibitor vorinostat increased GD2 expression and synergised with anti-GD2 monoclonal antibody therapy, resulting in improved tumour control and survival (102). This approach, in combination with ^131^I-Metaiodobenzylguanidine (^131^I-MIBG) systemic radiotherapy, has been successfully used in a phase I study (103) and now reached phase II for relapsed/refractory NB (NCT07261241). These findings support the potential of epigenetic priming to increase antigen density and enhance anti-GD2 activity.

Current clinical efforts instead seek to improve anti-GD2 activity through immune-effector augmentation and multimodal combinations. These include rhIL-15 plus dinutuximab and irinotecan/temozolomide (NCT06995872), expanded allogeneic TGFβi NK cells combined with dinutuximab-based chemoimmunotherapy (NCT06450041) (Table 2).

**TABLE 2:** Representative clinical trials aimed at enhancing the efficacy of GD2-directed therapies in relapsed or refractory neuroblastoma**.** List of representative clinical trials obtained from ClinicalTrials.gov.

Trial ID	Phase	Population	Intervention	Efficacy-enhancing strategy	Registry status
NCT01767194	Phase 2	Children and young adults with refractory, relapsed or progressive neuroblastoma	Irinotecan/temozolomide plus dinutuximab and GM-CSF; compared with a temsirolimus-containing arm	Combining chemotherapy-induced tumour injury with GD2-directed antibody-dependent cytotoxicity	Completed
NCT04560166	Phase 2	Patients ≥12 months with primary refractory high-risk neuroblastoma or first relapse	Naxitamab plus GM-CSF and irinotecan/temozolomide	Chemoimmunotherapy approach to enhance immune-mediated killing in chemotherapy-resistant disease	Terminated
NCT06995872	Phase 1	Children and young adults with relapsed or refractory neuroblastoma	rhIL-15 plus dinutuximab and irinotecan/temozolomide	Augments NK- and T-cell activity through IL-15 support to strengthen anti-GD2 antibody-dependent cytotoxicity combined with chemotherapy	Recruiting
NCT06450041	Phase 2	Patients with relapsed or refractory neuroblastoma	Expanded allogeneic TGFβ-imprinted NK cells plus dinutuximab, temozolomide, irinotecan and sargramostim	NK cells conditioned to resist TGFβ-mediated immunosuppression to reinforce chemoimmunotherapy	Recruiting
NCT03332667	Phase 1	Patients with relapsed or refractory, MIBG-avid neuroblastoma	^131^I-MIBG plus dinutuximab, with or without vorinostat	Combined targeted radiotherapy with GD2-directed immunotherapy plus vorinostat as radiosensitising and epigenetic-modulating agent	Completed
NCT07261241	Phase 2	Patients with relapsed or refractory, MIBG-avid neuroblastoma	Randomised comparison of^131^I-MIBG plus vorinostat,^131^I-MIBG plus dinutuximab, or the triple combination	Compare radiopharmaceutical, anti-GD2 and HDAC-inhibitor combinations and define the contribution of each component	Not yet recruiting

Abbreviations: GM-CSF, granulocyte-macrophage colony-stimulating factor; HDAC, histone deacetylase; MIBG, metaiodobenzylguanidine; NK, natural killer; rhIL-15, recombinant human interleukin-15; TGFβ, transforming growth factor beta; TME, tumour microenvironment.

Beyond antibody-mediated cytotoxicity, GD2 can also be exploited as a targeting moiety for nanoparticle-based drug delivery. A recent preclinical study described human serum albumin nanoparticles functionalised with an anti-GD2 antibody to encapsulate the epigenetic compound PRT4165. This formulation enhanced cellular internalisation and induced apoptotic cytotoxicity in GD2-positive neuroblastoma cells, supporting the potential of combining antigen-directed delivery with epigenetic therapy (104).

Although GD2-targeted approaches are potentially applicable to all neuroblastomas, molecular profiling can uncover distinct oncogenic pathways that provide alternative vulnerabilities for pathway-directed precision therapies.

#### 4.1.2 ALK-directed therapies

As sensitivity to ALK inhibitors is mutation-specific, molecular characterisation of the individual ALK alteration is required to guide drug selection and clinical-trial enrolment.

*ALK* is a relatively common driver gene in both sporadic (105, 106) and familial (35) cases of high-risk NB, harbouring activating point mutations, amplifications or gene fusions. Despite different mutations demonstrating different levels of response to different inhibitors, ALK represents a promising druggable target in forms carrying *ALK* gene alterations. However, only a reduced number of these alterations are potentially actionable with the first-generation inhibitor crizotinib (105, 107). More promising are the next-generation inhibitors ceritinib and lorlatinib, which can also inhibit crizotinib-resistant alterations (108), thus reaching phase I and II clinical trials in *ALK* aberrant relapsed/refractory NB, both as monotherapy or in combination with standard chemotherapy (109, 110), with monotherapy efficacy limited by tumour heterogeneity and resistance to treatment (111).

Reflecting this clinical priority, the ACCELERATE Paediatric Strategy Forum actively prioritises targeted ALK inhibition. In particular, it supported a sequential, alteration-specific development of ALK inhibitors in NB, with particular attention to compounds active against resistant kinase-domain variants and to rational combination approaches (112).

Beyond direct kinase inhibition, ALK represents an ideal surface target for antibody-drug conjugates because its physiological expression is restricted to the developing nervous system (113, 114). Furthermore, since ALK mediates the activation of ERK, also targeting this protein, and consequently the MAPK signalling pathway, could be a promising therapeutic strategy (115).

Recent preclinical models highlight a synergistic cross-talk between splicing inhibition and protein degradation. Co-treating MYCN-amplified/ALK^F1174L^ neuroblastoma mouse model with the ALK inhibitor alectinib (which cross-inhibits the splicing regulator SRPK1) and the molecular glue indisulam (an RBM39 degrader), triggers R-loop accumulation and lethal DNA damage leading to complete tumour remission (116).

#### 4.1.3 MYCN-associated vulnerabilities

Because direct therapeutic targeting of the MYCN oncoprotein is possible (111) but remains notoriously difficult, exploring its downstream molecular dependencies offers a powerful alternative for precision oncology. Given that *MYCN* amplification drives high TERT expression - which can also occur independently by genetic rearrangements - disrupting telomeric maintenance pathways represent a promising strategy for high-risk disease.

In particular, MYCN expression can be destabilised through Aurora kinase A inhibition. To this end, the small molecule inhibitor alisertib demonstrated in phase I and II clinical trials meaningful response rates and manageable toxicity in relapsed/refractory NB (117, 118). Similarly, inhibition of the BET protein family provides an indirect method to suppress MYCN transcription and, in turn, inducing anti-proliferative and pro-apoptotic effects (119, 120).

Also inhibiting ornithine decarboxylase 1 (ODC1), thus limiting the polyamine biosynthesis necessary for MYCN activity, represents an additional strategy to target MYCN-driven NB. It has been found that difluoromethylornithine (DFMO), an FDA-approved ODC1 inhibitor used for trypanosomiasis, is able to suppress neuroblastoma cell-line proliferation (121), thus reaching phase I clinical trials as combined chemotherapy (122). DFMO has also progressed beyond early-phase evaluation as a post-immunotherapy maintenance strategy. In the single-arm phase II NMTRC003B study, patients with high-risk NB who had completed multimodal therapy, including anti-GD2 immunotherapy, received oral DFMO for up to 2 years. The treatment with DFMO was associated with improved event-free survival (hazard ratio, 0.50; 95% CI, 0.29–0.84; P = 0.008) and overall survival (hazard ratio, 0.38; 95% CI, 0.19–0.76; P = 0.007) (123). Although these data derive from a non-randomised comparison and require cautious interpretation, they provided the basis for the 2023 FDA approval of eflornithine to reduce relapse risk in adult and paediatric patients with high-risk NB who achieved at least a partial response after prior multimodal therapy (124). Consequently, DFMO represents one of the most clinically mature approaches targeting a MYCN-associated metabolic dependency and its integration into prospective frontline protocols is a top priority for upcoming trials.

Additional MYCN-dependent metabolic vulnerabilities offer innovative opportunities for precision intervention. GLDC, a key enzyme of the glycine-cleavage system, is a direct transcriptional target of MYCN and is highly expressed in *MYCN*-amplified neuroblastoma. It has been demonstrated that GLDC depletion impairs the proliferation and tumorigenicity of *MYCN*-amplified neuroblastoma cell lines by disrupting purine and central carbon metabolism, therefore possibly constituting a targeted therapeutic strategy in NB patients with *MYCN* amplification (72).

Finally, since *MYCN* amplification leads to replication stress, inducing synthetic lethality represents a highly potent complementary approach. This strategy can be exploited through the inhibition of ATR kinase (125) or PLK4 (126), both of which are essential to manage the DNA damage response. Similarly, targeting CHK1, either alone or in combination with WEE1, can trigger mitotic catastrophe and subsequent apoptosis (127, 128). Reflecting the potential of this strategy, combining the WEE1 inhibitor adavosertib with irinotecan has shown encouraging results and advanced to phase II clinical trials (129).

Ultimately, these pathway-directed agents should be viewed not as isolated monotherapies, but as modular components of rational combination strategies (90). GD2-directed strategies, ALK inhibition and MYCN-associated vulnerabilities represent distinct but possibly complementary therapeutic axes in high-risk NB, requiring precise molecular characterisation of each NB patient. Currently, GD2-directed antibody therapy has the greatest clinical maturity, whereas ALK inhibitors depend on alteration-specific patient selection. In contrast, most MYCN-targeted approaches remain investigational, with the exception of the ODC1 inhibitor DFMO, which represents the most clinically advanced metabolic dependency strategy to date.

### 4.2 Cell-based therapies

Recent advances in cellular immunotherapy have introduced promising treatment strategies for high-risk NB (130), mostly consisting in Chimeric antigen receptor T-cell (CAR-T) or natural killer (NK) cells based approaches, while additional platforms remain at earlier stages of development. Among them, CAR-T-cell therapy represents the most extensively investigated platform and has shown encouraging clinical activity (Table 3).

**TABLE 3:** Cellular immunotherapy approaches for high-risk neuroblastoma.

Strategy	Target	Key mechanism/advantage	Development stage	Clinical trial/evidence
**GD2-CART01 (third-generation CAR-T)**	GD2	Inducible caspase-9 safety switch with durable antitumour activity	Phase 1/2 clinical	ORR ∼66%, CR up to 40%, persistence >1 year in some patients and reported 5-year OS of ∼42–68% (131,132)
**Armoured CAR-T cells**	Mainly GD2	Cytokine secretion (IL-12/IL-15/IL-21) to counter immunosuppression and recruit immune cells	Preclinical/early translational	Supported mainly by preclinical and translational evidence (133)
**Costimulatory CAR designs**	Mainly GD2	CD28/4-1BB signalling to improve persistence and reduce exhaustion	Clinical/translational development	Supported by mechanistic and translational studies; represents a CAR-design optimisation rather than a distinct product class (134)
**GD2–B7-H3 SynNotch CAR-T**	GD2 priming + B7-H3 effector target	Dual-antigen logic gating improves specificity and limits off-tumour toxicity	Preclinical	Enhanced specificity and antitumour activity demonstrated in metastatic neuroblastoma xenograft models (135)
**CAR-T combined with MIF degradation**	GD2-directed CAR-T + MIF/TME	MIF degradation remodels the TME and restores CAR-T activity	Preclinical	Improved CAR-T function and tumour control demonstrated *in vitro* and *in vivo* (136)
**GD2-targeted CAR-NK cells**	GD2	Potent cytotoxicity, lower CRS/GVHD risk and off-the-shelf potential	Preclinical/early translational	Emerging alternative to CAR-T-cell therapy in neuroblastoma (137)
**Adoptive NK-cell therapy**	Multiple tumour ligands	Allogeneic/haploidentical NK cells with IL-2/IL-15 support to enhance cytotoxicity	Early clinical	Clinical studies have reported antitumour activity, particularly in combination regimens (138)
**Engineered γδ T cells and TILs**	Multiple tumour antigens	Complementary immune-recognition platforms with potential synergy with CAR/NK approaches	Early development	Proof-of-concept and translational evidence; dedicated neuroblastoma-specific clinical validation remains limited

Abbreviations: CAR, chimeric antigen receptor; CR, complete response; CRS, cytokine release syndrome; GVHD, graft-versus-host disease; MIF, macrophage migration inhibitory factor; NK, natural killer; ORR, overall response rate; OS, overall survival; PROTAC, proteolysis-targeting chimera; TILs, tumour-infiltrating lymphocytes; TME, tumour microenvironment.

#### 4.2.1 Chimeric antigen receptor T-cell (CAR-T) therapy

NB tumours often exhibit low mutational burden and limited neoantigen expression, which reduces spontaneous T-cell mediated immune recognition. However, they highly express specific cell-surface molecules such as disialoganglioside GD2 (131), making them amenable to targeted immunotherapy. CAR-T therapy involves engineered T cells designed to specifically recognise and kill tumour cells expressing GD2, offering a promising avenue for treatment-resistant or relapsed high-risk NB (131). GD2-targeting CAR-T cells (GD2-CART01) represent a third-generation CAR-T product that has demonstrated encouraging efficacy and manageable safety profiles in paediatric patients. Clinical trials have reported high overall response rates, with some studies indicating response rates of about 66% and complete remission rates up to 40% at various follow-up points. The therapy showed persistence in patients' blood for over a year in some cases, supporting long-term tumour control. Treatment-related toxicities such as cytokine release syndrome (CRS) and immune effector cell-associated neurotoxicity syndrome were relatively manageable, with safety mechanisms like an inducible suicide gene being effective in controlling adverse events. The overall survival rate after 5-year of GD2-CART01 treatment reached around 42%–68%, depending on patient selection, which is a notable improvement for this difficult-to-treat population (131, 132). Recent advancements also explore combination therapies to enhance CAR-T effectiveness, such as the co-administration of ALK inhibitors alongside ALK-targeted CAR-T cells, which has shown promise in preclinical models of metastatic neuroblastoma. These innovative approaches aim to overcome challenges posed by tumour heterogeneity and the immunosuppressive tumour microenvironment that limit CAR-T cell efficacy (132). Overall, GD2 CAR-T therapy holds great promise as a novel and effective approach for high-risk NB, offering durable remissions and improved survival outcomes, with ongoing trials aiming to optimise dosing, safety, and combination strategies to further improve patient prognosis.

Despite their potential, the efficacy of CAR-T cells in solid tumours is still limited by significant obstacles, including poor persistence, T-cell exhaustion, and an immunosuppressive tumour microenvironment. In particular, polymorphonuclear myeloid-derived suppressor cells (PMN-MDSCs) represent a clinically relevant mechanism of resistance to GD2-CAR-T-cell therapy. In fact, peripheral PMN-MDSC frequencies increased at relapse or loss of response and inversely correlated with circulating GD2-CAR-T-cell levels in patients with NB receiving this therapy. Moreover, the cytotoxic activity of GD2-CAR-T cells has been observed to be impaired by PMN-MDSC co-cultured *in vitro*, thus supporting their potential role as biomarkers of resistance and as targets for combination strategies (139, 140). Clinical experience has shown that T cell persistence is a substantial barrier, with many studies reporting suboptimal durability and potency of CAR-T cells in solid tumours compared to haematologic malignancies (141). Specifically, early-phase clinical trials involving Epstein-Barr virus (EBV)-specific GD2 CAR-T cells, the engineered T cells persisted on average for about 6 weeks. Despite some patients achieving partial or complete tumour responses, many ultimately experienced disease progression, which was attributed to the limited long-term expansion and durability of the CAR-T cells (142).

Recent innovations address these limitations by incorporating costimulatory domains to enhance T cell survival and functionality or engineered to secrete cytokines improving tumour infiltration. Strategies to enhance CAR-T cell trafficking and infiltration into tumour sites - crucial for targeting solid tumours like neuroblastoma - are also in development. Combination therapies pairing CAR-T cells with immune checkpoint inhibitors or targeted agents are under active investigation to overcome resistance mechanisms and sustain antitumour responses effectively. The incorporation of costimulatory domains such as CD28 and 4-1BB into CARs has been pivotal in enhancing T cell survival, proliferation, and antitumour activity. These costimulatory signals promote sustained CAR-T cell expansion and resistance to exhaustion, significantly improving the durability and effectiveness of the response (134). Additionally, armoured CAR-T cells designed to secrete cytokines such as IL-12, IL-15, or IL-21 locally within the tumour microenvironment have shown promise in overcoming immunosuppression. By releasing pro-inflammatory cytokines, these armoured CAR-T cells modulate the tumour milieu to favour immune activation and enhance infiltration. This strategy not only boosts CAR-T cell function but also recruits and activates endogenous immune cells, amplifying the antitumour effect (133).

Enhancing CAR-T cell trafficking and infiltration remains another key area of development. Approaches include engineering CAR-T cells to express chemokine receptors that match the chemokines secreted by tumours, thereby improving homing to tumour sites. Modifying adhesion molecules and combining CAR-T therapy with agents that alter the extracellular matrix are also under exploration to facilitate deeper tumour penetration (143). Furthermore, combination therapies pairing CAR-T cells with immune checkpoint inhibitors (such as anti-PD-1 or anti-CTLA-4 antibodies) or targeted agents aim to overcome resistance mechanisms (144). These combinations address T cell exhaustion and the immunosuppressive tumour microenvironment, fostering sustained CAR-T cell activity and more durable clinical responses. All these encouraging results have propelled the development of multi-centre Phase II trials aimed at expanding access and refining combinatorial regimens to overcome tumour immune evasion.

Additionally, innovative strategies combining CAR-T cells with novel agents such as macrophage migration inhibitory factor (MIF) inhibitors are being actively explored in preclinical studies, aiming to enhance tumour vulnerability to immune attack and improve CAR-T therapeutic efficacy. In a recent investigation, authors demonstrated that targeting MIF using advanced PROTAC technology to degrade the protein leads to a significant enhancement in CAR-T cell function and tumour control in both *in vitro* and *in vivo* models. This approach effectively disrupts the tumour’s immunosuppressive microenvironment, restoring immune activation and promoting sustained antitumour responses (136). These promising findings support the development of this combination therapeutic approach for clinical trials, potentially providing a new avenue to overcome immune resistance in high-risk NB (136).

Next-generation logic-gated CAR-T-cell platforms may further improve the specificity and safety of GD2-directed cellular therapy. In the GD2–B7-H3 SynNotch system, recognition of GD2 acts as a priming signal that induces the expression of a B7-H3-directed CAR, restricting full CAR activation to tumour cells co-expressing GD2 and B7-H3. This platform design has the potential to reduce off-tumour activity against cells that express only one of the two antigens, making it potentially safer and more effective than traditional CAR-T approaches. These gated CAR-T cells displayed enhanced tumour specificity and antitumour activity *in vitro* and in metastatic xenograft models of neuroblastoma, together with lower expression of exhaustion markers and improved oxidative metabolic fitness compared with conventional B7-H3 CAR-T cells (135). Although highly promising, this approach remains preclinical and requires further evaluation.

These innovations represent a paradigm shift in CAR design and improving T cell functionality specifically tailored to tackle the unique challenges posed by NB. These advancements are driving a new era of cellular immunotherapy, offering renewed hope for significantly improved outcomes in this challenging paediatric malignancy. As clinical trial data continue to mature and validate these approaches, their integration into standard care protocols is increasingly anticipated, potentially transforming the therapeutic landscape and survival prospects for high-risk NB patients.

#### 4.2.2 Other cell-based therapies

NK cell-based therapies constitute a promising and rapidly evolving avenue in the treatment of high-risk NB, primarily due to their inherent advantages such as a reduced risk of graft-versus-host disease and generally lower toxicity profiles compared to T cell–based therapies. Clinical studies have demonstrated that adoptive transfer of expanded NK cells, including both allogeneic and haploidentical sources, can achieve significant antitumour activity, particularly when combined with cytokine support such as interleukin-2 (IL-2) or interleukin-15 (IL-15) which enhance NK cell proliferation and cytotoxic function (138). In recent years, GD2-targeted CAR-NK cells have emerged as a highly promising alternative to CAR-T therapies. These genetically engineered CAR-NK cells exhibit potent cytotoxicity against NB cells while maintaining a safer side effect profile, including a markedly reduced risk for severe cytokine release syndrome, a common complication associated with CAR-T therapies (137). Furthermore, CAR-NK cells can be derived from multiple cellular sources, including peripheral blood, umbilical cord blood, and NK-92 cell lines, facilitating scalable manufacturing and potentially enabling “off-the-shelf” therapeutic products. Beyond NK platforms, additional cellular strategies such as engineered γδ T cells and tumour-infiltrating lymphocytes (TILs) remain in earlier phases of development but offer unique immunologic mechanisms that may complement or synergise with NK- and CAR-based treatments. Altogether, continued refinement of manufacturing, persistence-enhancing strategies, and integration with multimodal regimens positions NK cell immunotherapy as a compelling component of future therapeutic frameworks for high-risk NB.

## 5 Conclusion

Managing high-risk NB remains highly challenging due to locally advanced tumours, metastasis and frequent recurrence after conventional therapies. This makes the integration of personalised medicine into the treatment of high-risk NB a promising frontier in paediatric oncology.

Recent advances in high-throughput multi-omics have greatly expanded our understanding of the underlying mechanisms driving NB, facilitating the discovery of novel disease-associated biomarkers. These biomarkers refine patient stratification and contribute to the design of tailored therapeutic strategies.

Within this expanding molecular landscape *MYCN* amplification remains the most clinically significant tumour-specific indicator of aggressive disease, and, for this reason, it should be considered a central component of risk stratification. The additional genomic, transcriptomic, proteomic and metabolomic biomarkers discussed in this review serve as complementary variables that may refine risk prediction. When biologically actionable, these biomarkers reveal specific vulnerabilities and provide targetable candidates for precision strategies. Crucially, while multi-omics offer unprecedented insights, their clinical translation must balance financial costs and analytical turnaround times, prioritising accessible platforms like MRD monitoring via liquid biopsy.

Currently, innovative approaches including anti-GD2 strategies, ALK inhibitors and CAR-T cell therapies, have demonstrated promising results. The transition towards biomarker-driven combinations offers the opportunity to overcome drug resistance, improve survival rates and preserve the long-term quality of life for paediatric patients with high-risk neuroblastoma.
